# A novel disulfidptosis-related immune checkpoint genes signature: forecasting the prognosis of hepatocellular carcinoma

**DOI:** 10.1007/s00432-023-05076-4

**Published:** 2023-07-18

**Authors:** Yuxin Chen, Wanying Xue, Yuting Zhang, Yu Gao, Yuanyuan Wang

**Affiliations:** School of Life Science, Bengbu Medical College, Bengbu, Anhui China

**Keywords:** HCC, Immune checkpoints, Disulfidptosis, Prognosis signature model

## Abstract

**Background:**

HCC is an extremely malignant tumor with a very poor prognosis. In 2023, a brand-new kind of cell death known as disulfidptosis was identified. Although, the prognosis as well as expression of immune checkpoints that are closely connected with it in HCC remain unknown.

**Methods:**

In this work, we identified 49 genes with abnormal expression in liver cancer and normal liver tissue, with 23 of them being differentially expressed genes. To create a signature, we classified all HCC cases into three subtypes and used the TCGA database to evaluate each relevant gene’s prognostic value for survival.

**Results:**

Five gene signatures were identified using the LASSO Cox regression approach, while those diagnosed with HCC were split into either low- or high-risk groups. Patients having low-risk HCC showed a much greater likelihood of surviving than those with high risk (*p* < 0.05). Through immune cell infiltration analysis, it was found that immune-related genes were abundant in high-risk groups and had reduced immune status.

**Conclusion:**

In conclusion, immune checkpoint genes highly associated with disulfidptosis contribute to tumor immunity and can be used to evaluate HCC prognosis. When it comes to predicting overall survival (OS) time in HCC, risk score has been set to be a separate predictor. Through immune cell infiltration analysis, it was found that immune-related genes were abundant in high-risk groups and had reduced immune status. It is possible to measure the prognosis of HCC based on immune checkpoints genes strongly linked to disulfidptosis.

**Supplementary Information:**

The online version contains supplementary material available at 10.1007/s00432-023-05076-4.

## Introduction

Liver cancer still leads the most cause of cancer-related death worldwide (Villanueva 2019). It was predicted that in 2025, it will affect a total of 100,000 people (Xiao et al. 2022). Hepatocellular carcinoma (HCC) is the most common type of liver cancer, and it is estimated that approximately 25% of those who are afflicted by HCC have mutations (Song et al. 2019). HCC is the most commonly diagnosed primary cancer of the liver, and is responsible for up to 90% of all liver cancer cases (Wang et al. 2020). The treatment of HCC, which now includes hepatic resection and live transplantation, has significantly improved since the early 2010s (Galle et al. 2018; Miura et al. 2012). Following these potentially curative approaches, adjuvant therapies to preclude relapse are still un unmet medical. Thus, the need for credible novel prognostic models is urgent, as this will increase the viability of targeted therapies.

Cancer immunotherapy stimulates the immune system, which leads to cytotoxicity in cancer cells (Miura et al. 2012; Gao et al. 2022). Immune checkpoints inhibit the activation of T cells and natural killer cells, as well as the initiation and maintenance of tumor immune tolerance (Nihira and Miki 2022; Zhu et al. 2021). Checkpoint inhibitors (ICIs) are currently posing a threat to the use of traditional HCC treatments (Xia et al. 2022). In the last 5 years, this discipline has seen significant advancements in the development of therapies, with studies indicating a notable improvement in patient quality of life and overall survival (Llovet et al. 2018). By eliminating negative immune regulatory checkpoints using therapeutic antibodies, the “immune checkpoint blockade” against cancer activates an already present anti-tumor immune response (Kalbasi and Ribas 2020).

At the beginning of 2023, a novel form of cell death has been observed which appears different from ferroptosis and apoptosis, and hailed as disulfidptosis. Abnormal accumulation of disulfides in cells induces a stress response and is highly toxic to cells (Liu et al. 2020; Joly et al. 2020). The reduced form of NADPH (Nicotinamide adenine dinucleotide phosphate) maintains cell survival by reducing disulfide bonds. The authors discovered that disulfide stress triggers disulfidptosis and even proposed a unique strategy for targeting disulfides in cancer treatment (Liu et al. 2023).

According to the findings, immune checkpoints and disulfidptosis are crucial for the growth of tumors and the anti-tumor processes. However, the relevance between them and their particular roles in HCC have received little research. To assess the differentially gene expression between hepatocellular carcinoma (HCC) and normal tissues, we compared the expression of two sets of genes: immune checkpoints and disulfidptosis-related genes. Next, screen genes for immune checkpoints highly associated with disulfidptosis and investigate the prognostic significance of these. We created a prognostic model to observe the interaction between these genes and the tumor immune microenvironment.

## Materials and methods

### Data collection identification of disulfidptosis-related immune checkpoint genes

In April 2023, we obtained RNA sequencing and clinical characterization data from the TCGA datasets for 374 HCC patients and 5 normal tissues. Next, 79 immune checkpoint-related genes and 23 disulfidptosis-related genes were extracted from prior reviews (Hu et al. 2021), and they are displayed in Table S1. To compare the expression data, fragments per kilobase million (FPKM) values were converted. Next, immune checkpoint genes strongly associated with disulfidptosis were screened by correlation analysis. Two thresholds were used to achieve that goal: cor > 0.3, *p* < 0.05 indicates a positive correlation, while cor < − 0.3, *p* < 0.05 indicates a negative correlation. These genes were connected through a PPI network using the PPI Search Tool (STRING) (https://string-db.org/). The “limma” package was used to identify differentially expressed genes (DEGs) with a *p* value < 0.05, logFC > 0.5. Among them, 49 genes were screened out, we further identified 23 DEGs.

### Development and validation of the disulfidptosis-related immune checkpoint differentially expressed genes prognostic model

To further evaluate the prognostic value of these 49 genes, we conducted a Cox regression analysis. This analysis was conducted to explore the correlation between the gene expression and the survival status. For further analysis, 5 genes were identified using a cut-off *p* value of 0.05. Next, R package “glmnet” was utilized to construct the LASSO Cox regression model. It helps to create the prognostic model and to narrow down the candidate genes. To calculate the penalty parameter, we used the minimum criteria and retained these 5 genes and their coefficients.

TCGA data are first centrally standardized through the R package “scale”, then a Risk Score was calculated (Risk Score = ${\sum }_{i}^{7}Xi\times Yi$, *X*: coefficients, *Y*: gene expression level). HCC patients were divided into high- and low-risk groups based on median risk scores, and OS time between these two groups were compared by Kaplan–Meier analysis. In the final step, plot the PCA with the R package “status”, and “survminer” and “timeROC” to plot the ROC survival curve for 3 years.

### Nomogram establishment using risk score and clinical factors

We conducted a comprehensive investigation into the connection between clinical factors and the DEGs signature in HCC patients. Additionally, our analysis included univariate and multivariate Cox regression analysis in combination with other clinical variables. We aimed to study whether risk scores had an independent prognostic value in predicting the outcome. To construct a nomogram linked to outcome for assessing the likelihood of overall survival time, we obtained data from the clinical variables and the risk score. We then applied to Cox regression model to calculate the hazard ratio (HR) and 95% confidence interval (CI) of each variable.

### Immune cell infiltration analysis

Our research aimed to investigate the differences in immune cell infiltration between risk groups. We employed the CIBER-SORT, CIBERSORT-ABS, QUANTISEQ, MCP-counter, XCELL, TIMER, and EPIC algorithms. The data obtained from these algorithms were then compared and analyzed. After gathering the data from the study, we conducted a comparative analysis of immune cell infiltration and immune pathway activation for each patient.

### Statistics analysis

Statistical analyses were performed using R software, which was used for all of the data analysis. The Pearson chi-square test was conducted the assess the differences between categorical variables, while single-factor analysis of variance was utilized to analyze the differences between normal liver and HCC samples. We used the Kaplan–Meier method along with a two-sided long-rank test to compare the OS between subgroups. For comparison across subgroups of patients, we used Kaplan–Meier approach and a two-sided log-rank test.

## Results

### Identification of disulfidptosis-related immune checkpoint genes between normal and tumor tissues

**Fig. 1:**
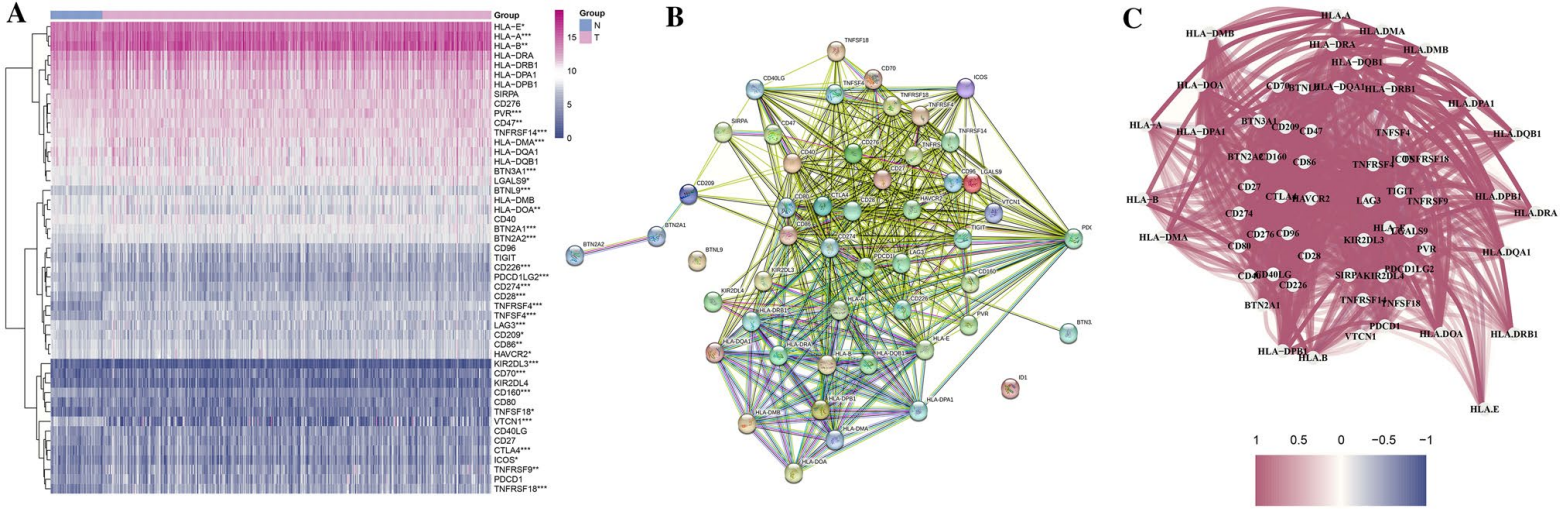
**A** A heatmap depicting the expression of the disulfidptosis-related immune checkpoints between normal and tumor tissues is presented. In this heatmap, purple indicated a high level, while blue indicates a low expression level. *p < 0.05, **p < 0.01, ***p < 0.001. **B** A PPI network of disulfidptosis-related immune checkpoints was constructed, which includes the interaction of these genes with interaction score of 0.9. **C** The correlation network of the disulfidptosis-related immune checkpoints shows a complex relationship between different factors. The purple lines indicate positive correlations, while the blue lines indicate negative correlations

The expression levels of immune checkpoint-related genes and disulfidptosis-related genes in hepatocellular carcinoma (HCC) were compared in 50 normal tissues and 374 tumor tissues in The Cancer Genome Atlas (TCGA) dataset, then 67 and 23 genes were, respectively, identified. Next, immune checkpoint genes strongly associated with disulfidptosis were screened by correlation analysis. Two thresholds were used to achieve that goal: cor > 0.3, *p* < 0.05 indicates a positive correlation, while cor < − 0.3, *p* < 0.05 indicates a negative correlation. Among them, 49 genes were screened out, we further identified 23 differentially expressed genes (DEGs) (all *p* < 0.05, logFC > 0.5) and constructed its expression matrix as well as correlation network (Table S1). Figure 1A is a heatmap that presents the expression levels of 49 genes. The heatmap provides a visual representation of which genes are highly and lowly expressed, allowing for further analysis of gene expression. This study explored the connections between these disulfidptosis-related immune checkpoint genes by analyzing their protein–protein interactions (PPIs) (Fig. 1B). This analysis set the minimum required interaction score at 0.9 (the highest confidence), and the correlation network of these genes was presented in Fig. 1C. Among them, we determined that these genes were strongly positive correlations.

### Tumor classification based on the DEGs

**Fig. 2:**
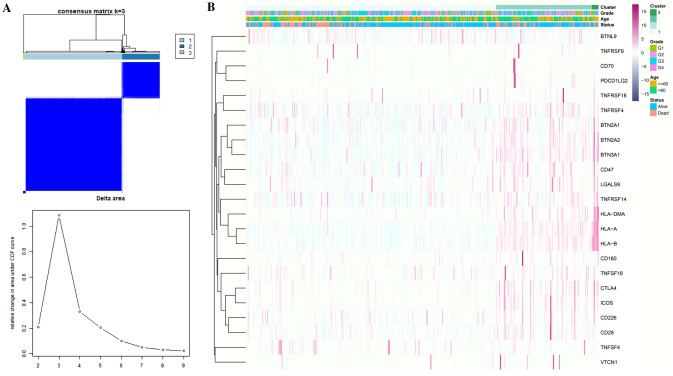
**A** The consensus clustering matrix (*k* = 3) was used to divide 374 HCC patients into three clusters. **B** These DGEs are characterized by a heatmap and clinicopathologic characteristics to identify two cluster (G1: high differentiation, G2: moderate differentiation, and G3: poor differentiation)

All 374 HCC patients in the TCGA cohort were used in a consensus clustering analysis to investigate the relationships between the expression of the 23 disulfidptosis-related immune checkpoints differentially expressed genes (DEGs) and HCC subtypes. The patients could be efficiently divided into three groups based on the 23 DEGs when this clustering factor (K) was placed at three. At this time, the correlation inside these three groups is the strongest, while the correlation between them is the weakest. (Fig. 2A).

The degree of tumor differentiation (G1–G3), age (≤ 60 or > 60 years), and survival status (alive or dead) were exhibited along with the gene expression profile in a heatmap, and we discovered that there were few changes in the clinical characteristics between this three groups (Fig. 2B).

### Construction of a disulfidptosis-related immune checkpoint prognostic gene model in TCGA

**Fig. 3:**
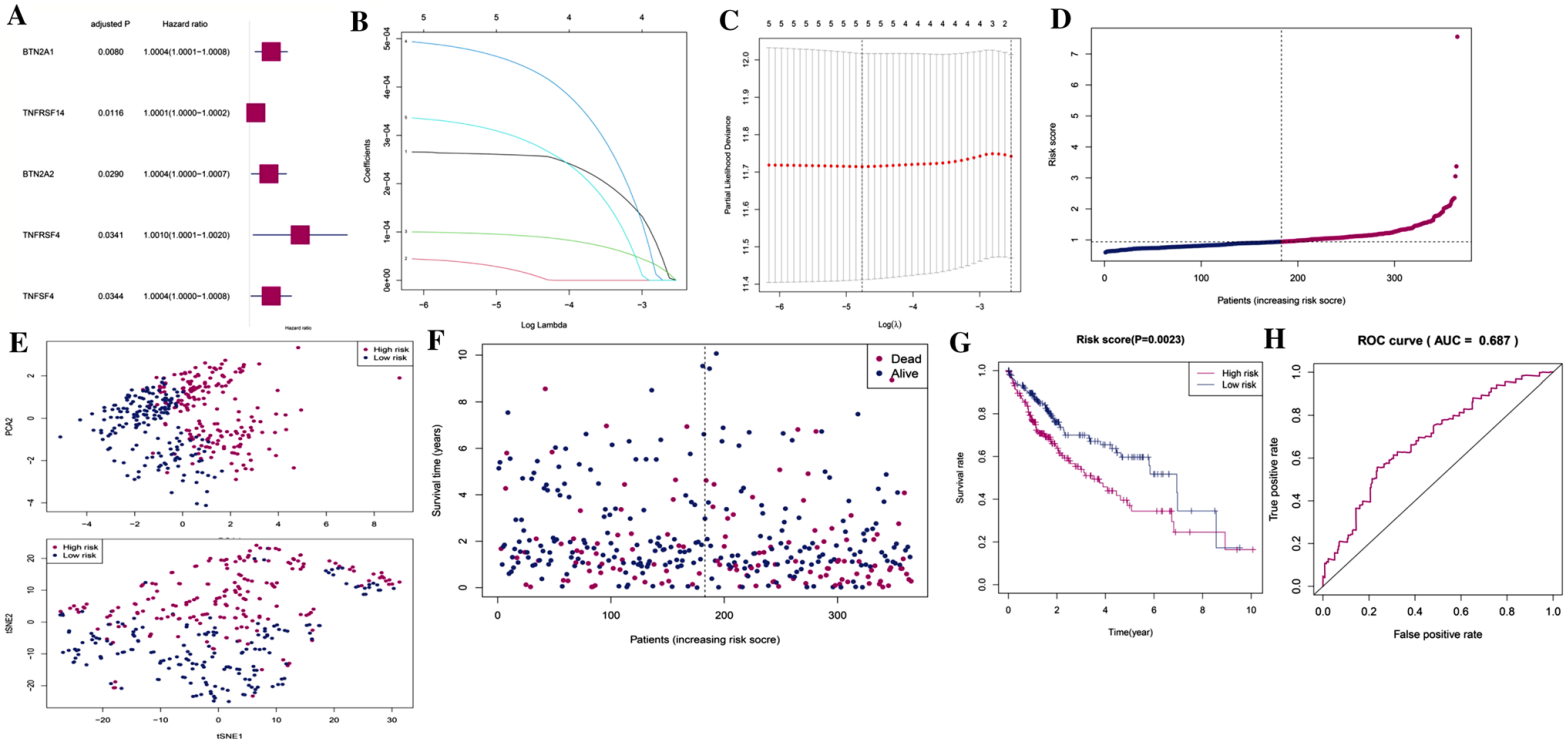
**A** Univariate Cox regression analysis of HCC for each disulfidptosis-related immune checkpoint prognostic gene, and 5 genes with *p* < 0.05. **B** LASSO regression of the 5 disulfidptosis-related immune checkpoint prognostic genes. **C** Cross-validation is used in the LASSO regression. **D**, **E** An analysis of the risk scores of HCC patients can be conducted through PCA and t-SNE plots. **F** It shows a statistically significant differences in mortality rate between high- and low-risk populations are shown in the figure below. **G** The Kaplan–Meier curves for HCC patients were analyzed to compare the survival rates between high and low-risk groups. **H** Risk scores' predictive effectiveness was shown via ROC analysis

To screen for genes related with survival, a univariate Cox regression analysis was preliminarily utilized. The 5 genes (BTN2A1, BTN2A2, TNFRSF14, TNFRSF4, TNFSF4) that fulfilled the *p* < 0.05 threshold were maintained for further analysis, and these genes were linked to higher risk with HRs > 1 (Fig. 3A). A five gene signature was created using the least absolute shrinkage and selection operator (LASSO) Cox regression analysis. This analysis was conducted to identify the ideal λ value that would yield a reliable gene signature. (Fig. 3B, C). With the help of these five genes, a risk model was successfully built. The following relevant coefficients were used to determine the risk score: risk score = (0.0002652 × BTN2A1 expression) + (0.00005213 × BTN2A2 expression) + (0.0001025 × TNFRSF14 expression) + (0.0005075 × TNFRSF4 expression) + (0.0003501 × TNFSF4 expression) (Concordance index = 0.606). According to the median risk score, 374 HCC patients were split into two equal subgroups, a low-risk group and a high-risk group. This split allowed for further analysis to better understand the differences between them (Fig. 3D). Next, we used the principal component analysis (PCA) and T-Distributed Stochastic Neighbor Embedding (tSNE). The PCA showed that the two groups were characterized by different combinations of risk factors, with high-risk group having a higher overall risk than the other. The tSNE visualization of the data further highlighted the two distinct cluster of patients (Fig. 3E). In comparison to the low-risk group, the deaths in the high-risk group occurred more frequently and had shorter survival times (Fig. 3F). The results of our study revealed a significant difference in OS time between low and high-risk groups (*p* < 0.01, Fig. 3G). This indicates a negative correlation between the risk score and prognosis, suggesting that the higher risk scores are associated with poorer prognosis. The prognostic model was assessed for its sensitively and specificity using a time dependent receiver operating characteristic (ROC) analysis. The area under the prognosis accuracy curve (AUC) for a 5-year period was determined to be 0.678, indicating that the model was able to accurately predict outcomes with a high degree of accuracy (Fig. 3H).

### Association between the risk model and independent prognostic value

**Fig. 4:**
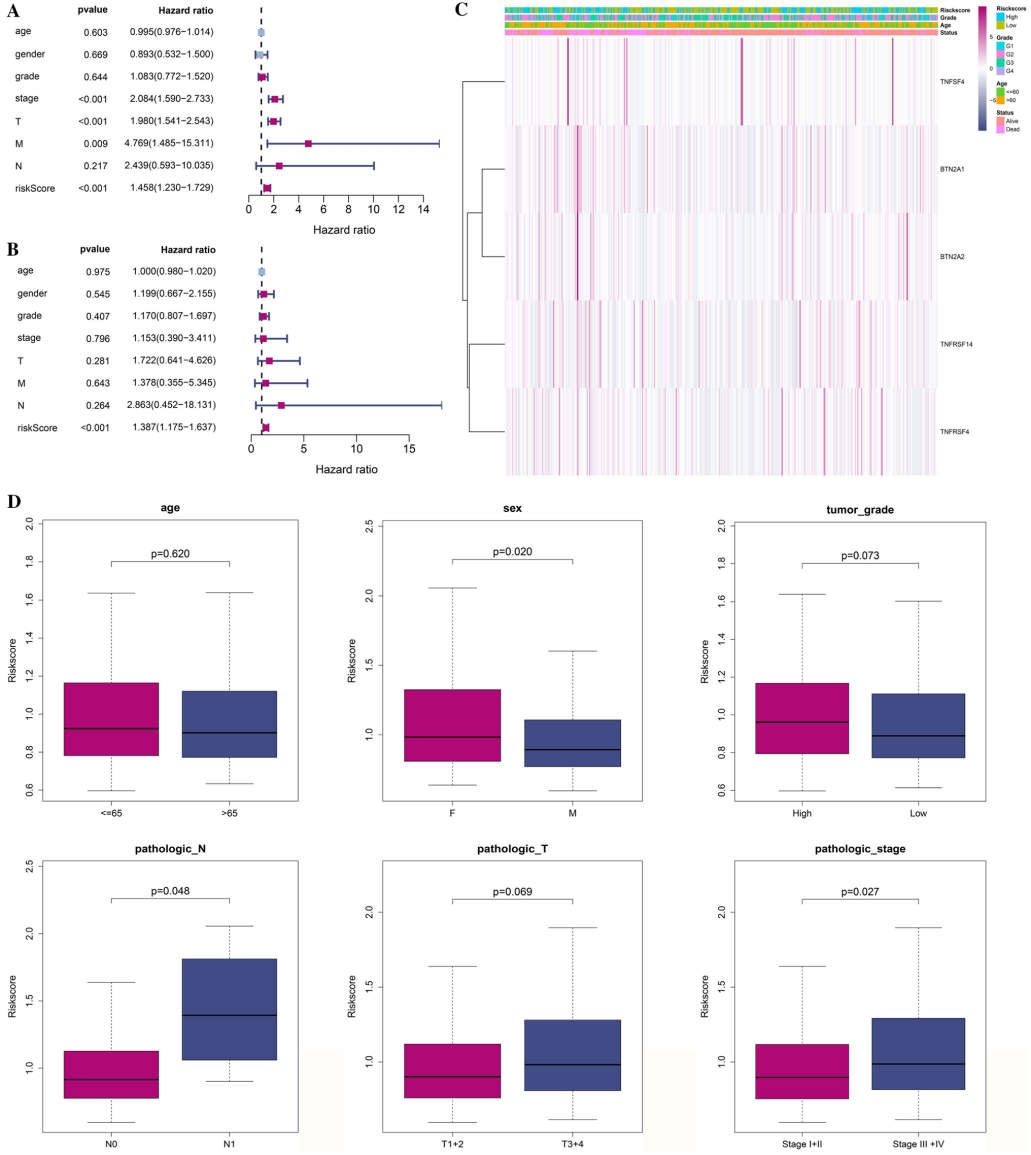
**A** The univariate Cox regression analysis revealed that there were connections between risk score for OS time and clinicopathological variables. **B** Multivariate Cox regression analysis was conducted to investigate the relationships between the risk score for OS time and clinicopathological variables. **C** Heatmap showing the relationships between the risk groups and the clinicopathologic variables (blue: low expression; purple: high expression) (*p < 0.05). **D** A strong correlation between gene signature and clinical characteristics

A univariate and multivariable Cox regression analysis was performed to investigate the potential of risk score obtained from the gene signature to function as an independent prognostic factor. This analysis was conducted to identify any significant relationships between the risk score and various clinical parameters (HR 1.458, 95% CI 1.230–1.729, Fig. 4A). In Fig. 4B, the multivariate analysis of both cohorts of patients with OC suggests that the risk score is an important prognostic factor, even after controlling for other confounding variables. This indicates that the risk score might be an important predictor of outcomes for these patients, as it is able to account for a range of variables that may influence the results of the study (HR 1.387, 95% CI 1.175–1.637). Additionally, when we created a heatmap of clinical variables, we observed that the age and survival status were widely spread. This indicates that age and survival status may be important factors in the clinical variables that were studied (*p* < 0.05) (Fig. 4C). The chi-square test was utilized to determine whether the prognostic signature had any to the emergence and progression in HCC. The results revealed that there were significant variations in sex (*p* < 0.05), pathologic N stage (*p* < 0.05), and pathologic stage (*p* < 0.05). but no discernible variations in the others. The chi-square test showed that there was a significant correlation between the prognostic signature and the emergence and progression of HCC, indicating that the prognostic signature did in fact contribute to the development. (Fig. 4D).

### Construction of a nomogram

**Fig. 5:**
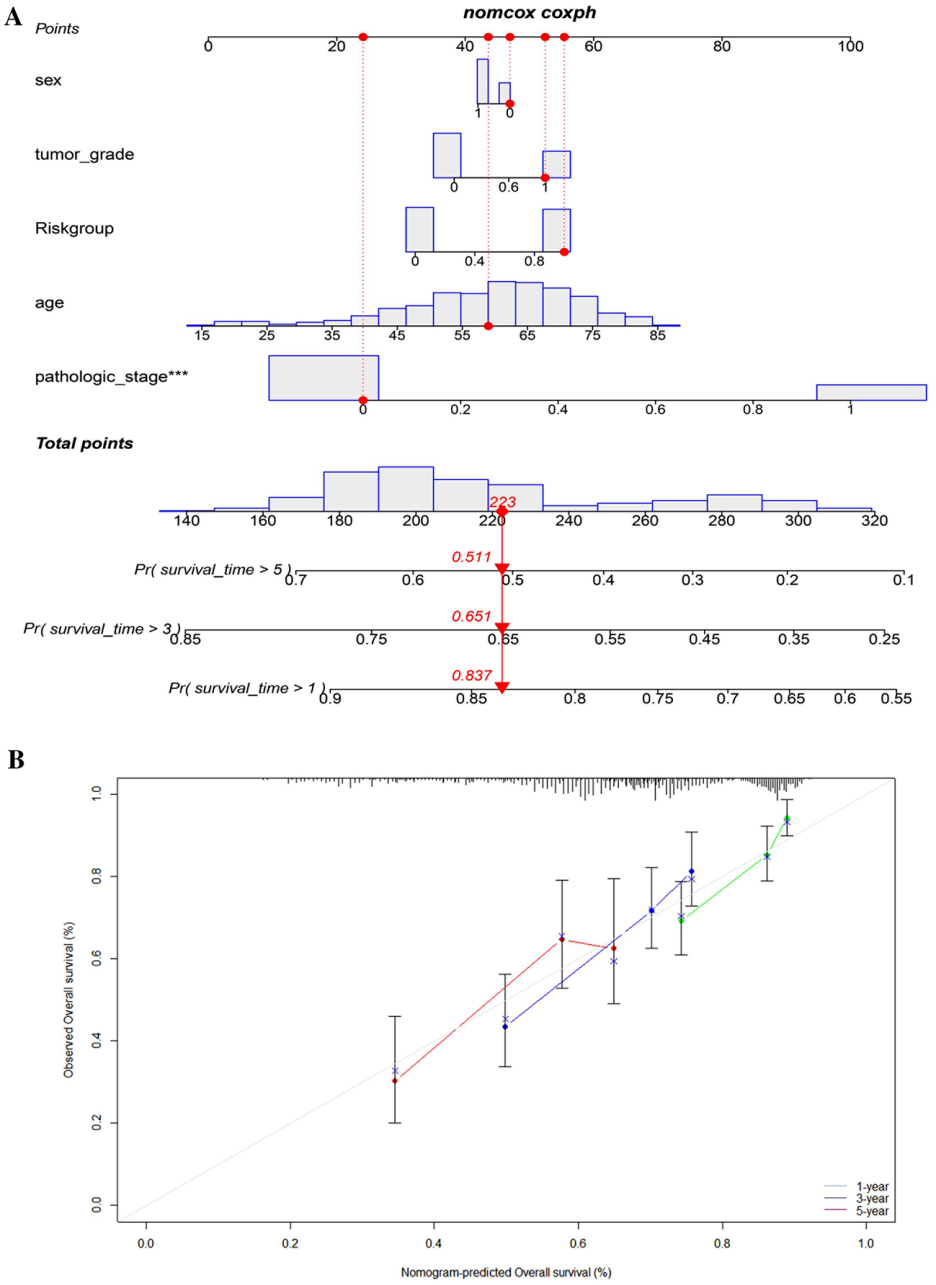
**A** Nomogram for predicting 1-, 3-, and 5-year OS. **B** The calibration plots for predicting 1-, 3-, and 5-year OS

The nomogram was designed to predict the overall survival of HCC patients. It included three different prognostic indicators: N stage, risk score and age. By plotting these three variables on this nomogram, medical professionals can accurately estimate the possibility of an individual’s survival. To further refine the prediction, the nomogram considered additional factors such as the patient's gender and comorbidities. The results detailed the patients' survival rates for HCC (Fig. 5A). These findings suggest that the prognosis for HCC patients is relatively good in the short term, with a gradual decline in survival rates over time. The results of the study also highlight the importance of early diagnosis and effective treatment for HCC patients, in order to maximize the chances of a positive outcome. The calibration curve in Fig. 5B revealed that the actual survival of the HCC patients matched the expected values.

### Functional analysis based on the risk model

Using the “limma” package, we were able to extract the differentially expressed genes (DEGs) based on the criteria of *p* < 0.01 and |log2FC |≥ 0.5. This gave us the opportunity to investigate the variations in gene functions and pathways between the subgroups identified by the risk model. Among them, 172 DEGs between the low- and high-risk groups in TCGA datasets were identified. To gain a better understanding of the biological pathways and functions associated with the differentially expressed genes (DEGs) identified, a gene ontology (GO) enrichment analysis and the Kyoto Encyclopaedia of Genes and Genomes (KEGG) pathway analysis were performed.

**Fig. 6:**
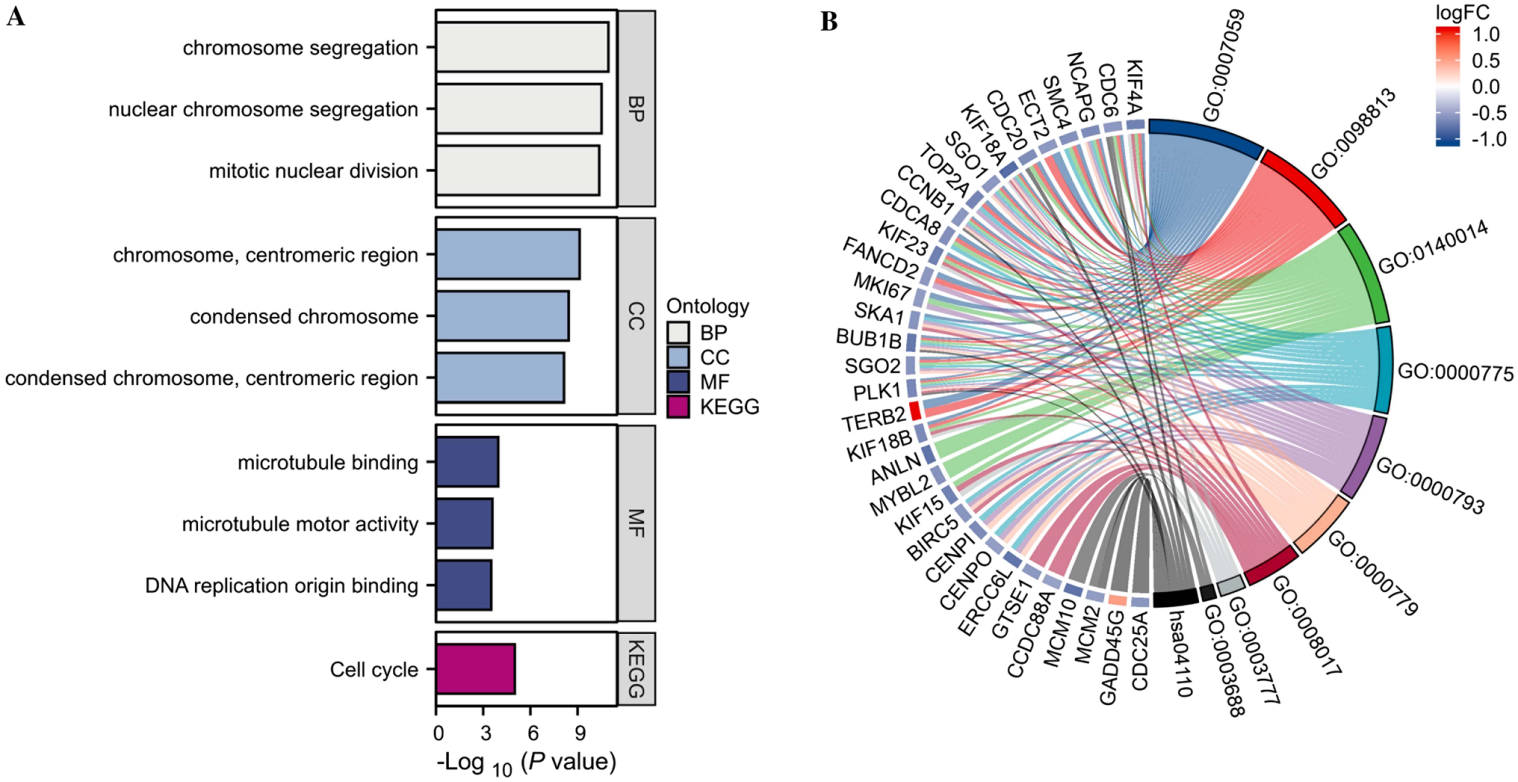
**A** A Bar plot showing for GO and KEGG pathways (longer bars indicate more enriched genes). **B** The GO and KEGG chord diagram of DEGs

A biological process analysis conducted recently revealed that a number of differentially expressed genes (DEGs) are significantly involved in the process of chromosome segregation, nuclear chromosome segregation and mitotic nuclear division. The cellular component analysis suggested that chromosome, centromeric region. Most enriched regions were on condensed chromosome and condensed chromosome, centromeric region. According to the molecular function analysis, DEGs were mainly located in microbubble binding, microtubule motor activity and DNA replication origin binding. In the KEGG pathway, the findings indicated that the cell-cycle signaling pathway was primarily linked with the DEGs (Fig. 6A). The results are also represented in the GO and KEGG chord diagram (Fig. 6B).

### Immune infiltration level and immune activity between risk model

**Fig. 7:**
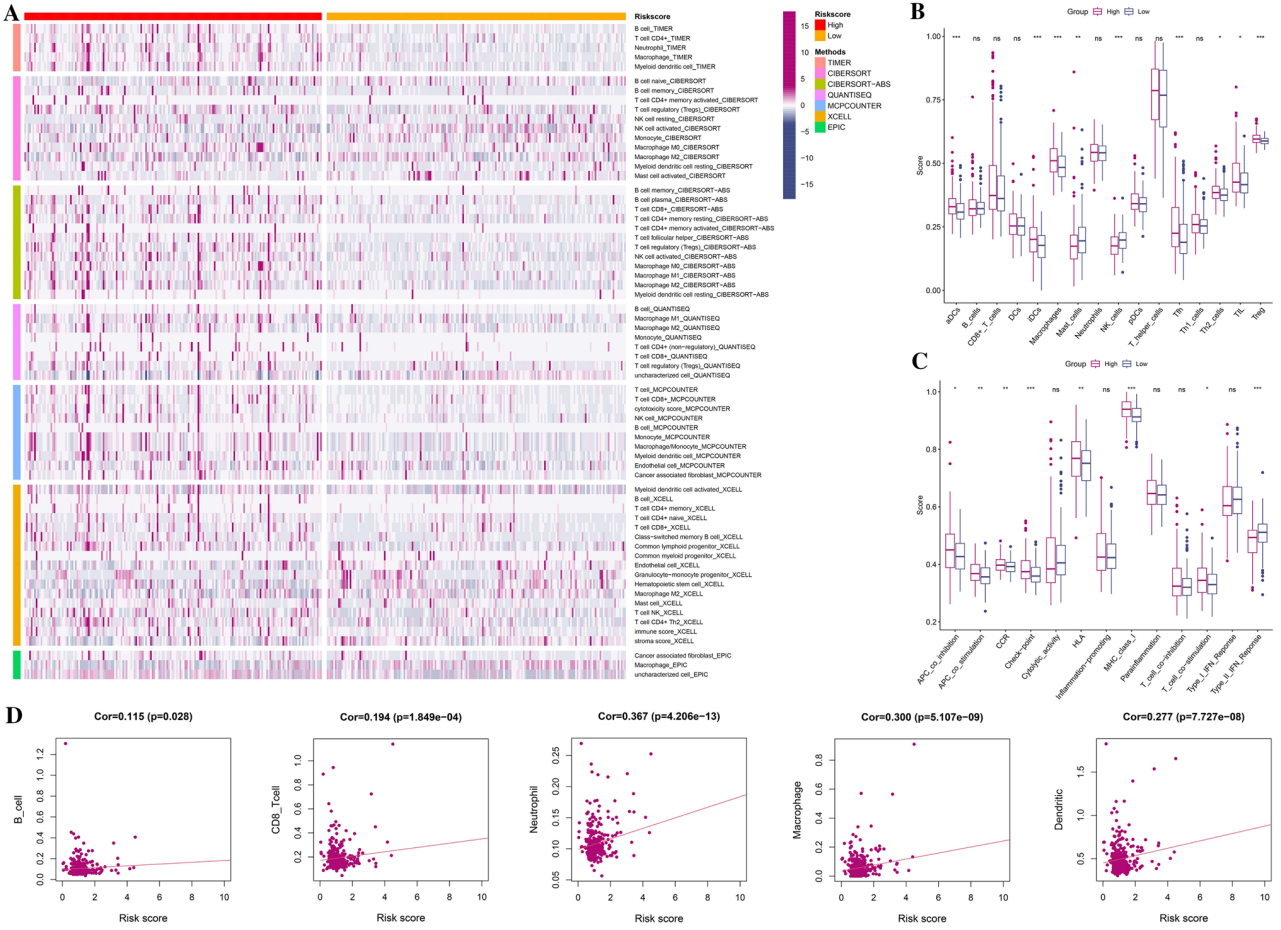
**A** The heatmap of immune cells infiltration between high-risk and low-risk groups. **B** The TCGA dataset enrichment scores of 16 types of immune cells were compared between low and high-risk group. **C** The TCGA dataset enrichment scores of 13 types of immune-related pathways were compared between low- and high-risk group. **D** A correlation between immune cell and risk score. *p < 0.05; **p < 0.01; ***p < 0.001

In Fig. 7A, the heatmap depicted the relationship between the signature and immune infiltration in accordance with the studies of TIMER, CIBERSORT, CIBERSORT-ABS, XCELL, QUANTISEQ, EPIC, and MCP-counter were shown as examples.

We conducted a simple-sample gene set enrichment analysis (ssGSEA) to develop a better understanding of enrichment scores across 16 immune cell types and pathways. This study was based on the functional analysis. The findings demonstrated that compared to the low-risk subgroup, the high-risk subgroup generally exhibited larger levels of immune cell infiltration, particularly of antibody–drug conjugates (aDCs), immature dendritic cells (iDCs), macrophages, natural killer (NK) cells, follicular helper T cell (Tfh), and regulatory cells (Treg). The other 12 immune pathways, with the exception of the type-2 IFN response pathway, were more active in the high-risk group than in the low one (Fig. 7B, C). According to Fig. 7D, immune cells are related to risk scores. The findings demonstrated that they had a clearly positive relevance in these immune cells with *p* < 0.05.

## Discussion

In this study, we first investigated mRNA levels in HCC and normal tissues of 67 and 23 currently known immune checkpoint genes and disulfidptosis-related genes, respectively. Next, immune checkpoint genes strongly associated with disulfidptosis were screened by correlation analysis; also, the PPI network of these genes was constructed. We found five genes with significant differences in expression and distinct differences in clinical features. We developed a risk model using Cox univariate analysis and LASSO Cox regression analysis to assess the prognostic value of certain outcomes. To further evaluate the performance of this model, we tested it on external datasets. What is more, both survival and ROC analysis showed that the signature had good predictive ability. Through functional analysis, we found that DEGs between low- and high-risk clusters were associated with cell-cycle pathway. Compared with the low-risk group, the high-risk group had universally increase in immune infiltrating cells as well as was positively correlated with most immune cells.

Disulfidptosis was a novel form of cell death newly discovered in early 2023, the mechanism of action is currently unclear. According to an article, the actin cytoskeleton's sensitivity to disulfide stress-mediated disulfidptosis may also have a role in cancer treatment by targeting disulfidptosis. It is still unclear how immune checkpoint genes strongly associated with disulfidptosis interact in HCC and whether this interaction is related to patient prognosis and survival. The five associated genes *TNFRSF14* (*HVEM*), *TNFRSF4* (*OX40*), *TNFSF4* (*OX40L*), *BTN2A1*, and *BTN2A2* that were discovered to predict OS time in HCC patients formed the signature that was created by our investigation. Tumor necrosis factor (*TNF*) ligands and cognate *TNF* receptor (*TNRF*) superfamilies combine to produce costimulatory signals that promote the survival, differentiation, and clonal expansion of CD4 + and CD8 + T cells in response to antigens (Pang and Qin 2022). It also has a significant impact on illness and T cell-mediated adaptive immunity. *TNFRSF* and *TNFSF* function as costimulatory receptors and ligands, respectively (Croft 2009; Croft et al. 2013). Many literatures have suggested that *TNFR* superfamily molecules emit costimulatory signals, are constitutive or inducible forms of T cells, and also play a significant role in protective immunity, inflammatory and autoimmune disease, and tumor immunotherapy (Coliță et al. 2023).

*TNFRSF14*(*HVEM*) is a subset of *TNF* receptor superfamilies (*TNFSF*) molecules that are widely expressed in many cell types, such as native and activated T cells (Kwon et al. 1997). In 2012, park found that *HVEM* promotes tumor-reactive T-cell response, induction and regression of long-term anti-tumor immunity (Park et al. 2012). *HVEM* cells contain three intact cysteine-rich domains (CRDs) outside the cell, and *LIGHT* can bind regions that span CRD2 and CRD3 (Bodmer et al. 2002; Gonzalez et al. 2005). When it binds to *LIGHT* as a ligand, it can trigger effective anti-tumor immunity that leads to tumor regression (Yu and Fu 2008). Kanodia discovered that its immunotherapy and antigen-specific vaccination combination was successful in clearing tumor-infiltrating C-D8 + T cell responses (Kanodia et al. 2010). These have demonstrated how potent anti-tumor immunity can be eliminated by *LIGHT*–*HVEM* interactions (Steinberg et al. 2011). A member of the tumor necrosis factor receptor family, *TNFRSF4* is also called *OX40* or *CD134* (Chen et al. 2022). It has 275 amino acids and is a type I transmembrane glycoprotein with three CRD domains and a partial C-terminal CRD (Fu et al. 2020). Its ligand, *OX40L* (*CD252*, *TNFSF4*), is a type II glycoprotein with a 133-amino acid extracellular domain that belongs to the *TNFRSF* (Croft 2010). They promote non-regulatory CD4 + and CD8 + T cell survival, maintain anti-apoptotic proteins BCL-XL, BCL-2 and BFL1 expression, increase cytokine production, enhance tumor-specific T cell immune responses, and increase tumor-specific memory T cell production after antigen challenge (Lee 2021).

More and more researches are being done on the function of *OX40* and its homologous ligand *OX40L* in immunomodulation in the treatment of cancer, particularly as a therapeutic target (Fu et al. 2020). The protein-coding gene *BTN2A1* (Butyrophilin Subfamily 2 Member A1) belongs to the immunoglobulin class. Among its related pathways are Innate Immune System and Class I MHC-mediated antigen processing and presentation. An important paralog of this gene is *BTN2A2*, a member of the BTN2 gene subfamily (Blazquez et al. 2018). They are all located on chromosome 6 and are involved in lipid, fatty acid and sterol metabolism. There are articles linking *BTN2A1* to poor prognosis for renal cell carcinoma and ovarian cancer (Billon et al. 2021; Fanale et al. 2022). Besides, Lebrero-Fernández C et al. found that *BTN1A1* and *BTN2A2* are associated with intestinal inflammation and cancer (Lebrero-Fernández et al. 2016). As a result, they might have a special function in various cancer types. In our study, these five genes were all highly expressed and significantly different in liver cancer, they all appeared to be cancer-promoting genes, and most of them were enriched in high-risk groups. The results of multivariate regression analysis are not always accurate, and univariate regression analysis shows a better performance against liver cancer. Therefore, how these genes interact in live cancer remains to be determined.

Research on disulfidptosis has been limited so far. Despite their strong correlation with disulfidptosis, these immune checkpoints may coexist and interact during tumor development. High levels of key tumor immunoinfiltrating cells indicate overall impaired immune function in the high-risk group. Surprisingly, Mast cell and NK cell (natural killer) had a statistically significant proportion of higher prognosis in the low-risk group than in the high-risk group, possibly linking them to anti-tumor immunity and adverse clinical outcomes.

Angélica Aponte-López et al. found that an increase in mast cell density was associated with a good or poor prognosis, depending on the tumor type and stage (Aponte-López and Muñoz-Cruz 2020). It not only regulated various events in tumor biology, such as cell proliferation, invasion and metastasis, tumor microenvironment, but also may shape the tumor microenvironment by establishing tandem interference with other tumor-infiltrating cells. Ombretta Melaiu et al. showed that natural killer cells are high cytotoxic, coordinated by complex inhibition and activation networks, and that high-density tumor infiltration is associated with a good prognosis for a variety of human solid tumors (Melaiu et al. 2019). These cells receive much less attention than other tumor-associated immune cells, but are now considered a key component of the tumor immune microenvironment and are expected to be potential targets for improved cancer immunotherapy. In light of the findings above, it might be due to a decrease in anti-tumor immunity that the high-risk group in HCC has a poor prognosis.

## Conclusions

In conclusion, we identified 49 genes with abnormal expression in liver cancer and normal liver tissue, with 23 of them being differentially expressed genes. Immune checkpoint genes highly associated with disulfidptosis contribute to tumor immunity and can be used to evaluate HCC prognosis. When it comes to predicting overall survival (OS) time in HCC, risk score has been set to be a separate predictor. Through immune cell infiltration analysis, it was found that immune-related genes were abundant in high-risk groups and had reduced immune status. It is possible to measure the prognosis of HCC based on immune checkpoints genes strongly linked to disulfidptosis.

## Supplementary Information

Below is the link to the electronic supplementary material.

Supplementary file1 (XLS 139 KB)

## Data Availability

TCGA belong to public databases. The patients involved in the database have obtained ethical approval. Users can download relevant data for free for research and publish relevant articles. Our study is based on open source data, so there are no ethical issues and other conflicts of interest.
